# Tumor and host characteristics of small cell lung cancer (SCLC) in U.S. community oncology practice

**DOI:** 10.1186/2051-1426-3-S2-P170

**Published:** 2015-11-04

**Authors:** Donald Richards, Si G Li, Thomas Anderson, Marc Monte, Brian Ulrich, David A Smith, Mahesh Seetharam, Paul Kaywin, Ray Page, Kimary Kulig, Cory Batenchuk, Maen Hussein

**Affiliations:** 1 US Oncology, Houston, TX, USA; 2 BioStat Solutions, Inc., Frederick, MD, USA; 3 Clopton Clinic of Jonesboro, Inc., Jonesboro, AR, USA; 4 Compass Oncology, Vancouver, WA, USA; 5 Arizona Oncology, Phoenix, AZ, USA; 6 Miami Cancer Institute, Baptist Health Medical Group, Miami, FL, USA; 7 The Center for Cancer and Blood Disorders, Fort Worth, TX, USA; 8 Bristol-Myers Squibb, Princeton, NJ, USA; 9 Florida Cancer Specialists, Tavares, FL, USA

## Background

The majority of lung cancer in the U.S. is treated in the community. A prospective cohort study of stage IV NSCLC and extensive disease (ED) SCLC is being conducted in 70 U.S. community practices to assess outcomes during the pre- and post-immunotherapy eras of lung cancer treatment. This analysis focuses on characteristics of the rarer SCLC subset in the pre-immunotherapy era.

## Methods

ED SCLC patients, at any point in their care, with documented dates of diagnosis and prior treatment are eligible. Patients are followed prospectively for 36 months or until death, with data abstraction from medical records. Archival tumor tissue (formalin-fixed paraffin-embedded) slides are used to measure PD-L1 protein expression with a validated, automated immunohistochemistry (IHC) assay from Dako using the 28-8 antibody. Expression levels of tumor cell membrane staining at any intensity were defined for ≥1% and ≥ 5% of 100 assessable tumor cells.

## Results

Data from 268 cases with complete records are reported. At enrollment, 95% were ever smokers, 53% female, and 26% ECOG performance status (PS) 2 or 3. The most prevalent sites of metastases were liver (34%), bone (32%), and brain (21%). History of a specific autoimmune condition was present in 8% of cases. The median overall survival from diagnosis with extensive disease will be reported. PD-L1 IHC was performed on 96/268 patients with available tissue and was evaluable in 87/96 cases. PD-L1 was expressed at ≥1% for 13 cases (15%) and ≥5% for 2 (2%) cases. Of these, 85 had hematoxylin and eosin (H&E) staining performed showing tumor inflammation in 62% and tumor necrosis in 41% of cases. An intra-tumoral inflammatory infiltrate of ≥10% was observed in 49% of cases and is associated with PD-L1 expression at ≥1% (*P* < 0.003).

## Conclusion

Many immunotherapy clinical trials exclude patients with active or untreated brain metastases and patients receiving treatment for autoimmune disease, yet a substantial proportion of community-based SCLC patients present with these attributes. PD-L1 expression levels in SCLC appear lower than those previously reported in NSCLC. The role of PD-L1 and other tumor and blood-based markers as prognostic or predictive for SCLC outcomes will be investigated in this ongoing study.

**Table 1:** Summary of clinical attributes and PD-L1 Expression in SCLC

Characteristic	Cohort	PD-L1 (<1%)	PD-L1 (≥1%)	Frequency in PD-L1 ≥1%	Fisher (pV)
Smoking status	EVER	70	13	15.7%	
	NEVER	4	0	0.0%	1.00

Sex	Male	40	6	13.0%	
	Female	34	7	17.1%	0.765

ECOG	0-1	49	7	12.5%	
	2-3	21	6	22.2%	0.335

Age	<70	47	5	10%	
	≥70	27	8	23%	0.126

Autoimmune Disorders	Present	4	3	42.9%	
	Absent	68	9	11.7%	0.057

Site of biopsy for PDL1 samples*	Primary	37	6	14.0%	
	Metastatic	36	7	16.3%	
	Primary/Metastatic	1	0	0.0%	1.000

H&E – Tumor Tissue Inflammation*	Absent	20	2	9.1%	
	Present	30	6	16.7%	0.6971

H&E – Tumor Tissue Necrosis	Absent	31	3	8.8%	
	Present	19	5	20.8%	0.2254

H&E-% Intra-Tumoral Inflammatory Infiltrate*	<10%	41	1	2.4%	
	≥10%	30	10	33.3%	0.003

